# Supplementary material for: The Effect of Low Glycemic Index Mediterranean Diet and Combined Exercise Program on Metabolic-Associated Fatty Liver Disease: A Joint Modeling Approach
Source: J Clin Med. 2022 Jul 26;11(15):4339. doi: 10.3390/jcm11154339 (PMC9330790; doi:10.3390/jcm11154339)
Supplement: Supplementary file 1 [file jcm-11-04339-s001.zip › jcm-1784103-supplementary.pdf]

**Supplementary Table S1. MAFLD diagnostic criteria over time**

| | Time of Follow-up (days) | | | | | |
|---|---|---|---|---|---|---|
| | Baseline | 45 | 90 | 180 | 270 | 360 |
| Whole Sample | | | | | | |
| N | 54 | 54 | 54 | 45 | 35 | 24 |
| **MAFLD** | 41 (74.1) | 37 (70.4) | 36 (66.7) | 31 (68.9) | 22 (62.9) | 17 (70.8) |
| Steatosis | 41 (74.1) | 38 (70.4) | 37 (68.5) | 31 (68.9) | 22 (62.9) | 17 (70.8) |
| **MAFLD1:** | | | | | | |
| BMI (kg/m$^2$) | 41 (74.1) | 37 (68.5) | 35 (64.8) | 30 (66.7) | 22 (62.9) | 17 (70.8) |
| **MAFLD3**: | | | | | | |
| Diabetes | 15 (36.6) | 15 (39.5) | 13 (35.1) | 10 (32.3) | 8 (36.4) | 5 (29.4) |
| Adherent Subjects | | | | | | |
| N | 39 | 39 | 39 | 31 | 25 | 18 |
| **MAFLD** | 30 (76.9) | 26 (66.7) | 26 (66.7) | 21 (67.7) | 15 (56.0) | 11 (61.1) |
| Steatosis | 30 (76.9) | 26 (66.7) | 27 (69.2) | 21 (67.7) | 15 (56.0) | 11 (61.1) |
| **MAFLD1:** | | | | | | |
| BMI (kg/m$^2$) | 30 (76.9) | 25 (64.1) | 25 (64.1) | 20 (64.5) | 15 (56.0) | 11 (61.1) |

| | | | | | | |
|---|---|---|---|---|---|---|
| **MAFLD3**: | | | | | | |
| Diabetes | 11 (28.2) | 10 (25.6) | 10 (25.6) | 6 (19.3) | 5 (20.0) | 4 (22.2) |

MAFLD: Metabolic-Associated Fatty Liver Disease; MAFLD1: MAFLD plus Body Mass Index (BMI) >25 kg/m2; MAFLD3: MAFLD plus Diabetes; Cells contain Frequencies (Percentage) calculated in relation to the number of subjects and may not add up to 100%.

**Supplementary Table S2**: Haematochemical, anthropometric and biomarker values over time.

| | Time of Follow-up (days) | | | | | |
|---|---|---|---|---|---|---|
| | Baseline | 45 | 90 | 180 | 270 | 360 |
| Whole Sample | | | | | | |
| N | 54 | 54 | 54 | 45 | 35 | 24 |
| BMI | 32,00 (5,14) | 31,06 (4,89) | 30,70 (5,00) | 30,68 (5,03) | 30,41 (5,34) | 31,95 (5,45) |
| SBP (mmHg) | 130,3 (11,9) | 122,8 (12,8) | 121,1 (11,6) | 125,9 (13,6) | 124,6 (10,1) | 126,5 (10,9) |
| DBP (mmHg) | 82,22 (8,22) | 78,24 (7,28) | 76,94 (6,62) | 79,78 (7,15) | 78,43 (6,84) | 80,21 (7,44) |
| GPT (UI/l) | 0,54 (0,28) | 0,45 (0,19) | 0,42 (0,16) | 0,44 (0,19) | 0,42 (0,24) | 0,39 (0,19) |
| GGT (UI/l) | 0,49 (0,50) | 0,38 (0,31) | 0,36 (0,27) | 0,39 (0,40) | 0,43 (0,44) | 0,37 (0,27) |
| GOT (UI/l) | 0,44 (0,14) | 0,40 (0,10) | 0,40 (0,10) | 0,40 (0,12) | 0,38 (0,12) | 0,35 (0,10) |
| TGL (mmol/L) | 1,74 (1,01) | 1,50 (0,96) | 1,48 (0,99) | 1,48 (0,69) | 1,49 (0,87) | 1,77 (1,24) |
| Chol-T (mmol/L) | 5,36 (1,01) | 5,07 (1,13) | 5,02 (0,96) | 5,09 (0,98) | 5,01 (1,10) | 5,28 (1,05) |
| HDL-C (mmol/L) | 1,15 (0,29) | 1,14 (0,29) | 1,15 (0,30) | 1,15 (0,26) | 1,17 (0,30) | 1,17 (0,33) |
| LDL-C(mmol/L) | 3,48 (0,61) | 3,44 (1,04) | 3,12 (0,83) | 3,16 (0,79) | 3,00 (0,93) | 3,27 (0,73) |
| Glucose (mmol/L) | 5,88 (1,57) | 5,70 (1,11) | 5,54 (0,98) | 5,65 (1,13) | 5,31 (0,66) | 5,44 (0,62) |
| HbA1c (mmol/mol) | 0,42 (0,10) | 0,41 (0,10) | 0,40 (0,08) | 0,38 (0,07) | 0,38 (0,07) | 0,37 (0,07) |
| HOMA-IR | 3,84 (2,34) | 3,18 (2,05) | 4,03 (9,49) | 3,73 (5,22) | 2,85 (1,94) | 3,09 (2,36) |
| Adherent Subj | | | | | | |
| N | 39 | 39 | 39 | 31 | 25 | 18 |
| BMI | 32,40 (5,31) | 31,37 (5,14) | 31,00 (5,34) | 30,72 (5,63) | 30,46 (6,05) | 32,18 (6,02) |
| SBP (mmHg) | 128,6 (11,1) | 121,8 (12,3) | 120,0 (10,5) | 124,0 (12,4) | 122,8 (10,6) | 123,6 (10,3) |

| | | | | | | |
|---|---|---|---|---|---|---|
| DBP (mmHg) | 81,41 (8,03) | 77,31 (6,96) | 75,77 (6,54) | 79,19 (6,60) | 77,80 (6,78) | 78,61 (7,44) |
| GPT (UI/l) | 0,52 (0,28) | 0,45 (0,21) | 0,41 (0,16) | 0,44 (0,21) | 0,43 (0,25) | 0,40 (0,22) |
| GGT (UI/l) | 0,55 (0,57) | 0,42 (0,35) | 0,39 (0,30) | 0,44 (0,48) | 0,48 (0,51) | 0,40 (0,31) |
| GOT (UI/l) | 0,43 (0,15) | 0,41 (0,11) | 0,39 (0,11) | 0,41 (0,13) | 0,38 (0,11) | 0,35 (0,10) |
| TGL (mmol/L) | 1,70 (0,85) | 1,45 (0,74) | 1,43 (1,02) | 1,43 (0,58) | 1,52 (0,83) | 1,69 (1,25) |
| Chol-T (mmol/L) | 5,35 (0,96) | 5,04 (1,04) | 5,02 (0,89) | 5,19 (0,89) | 5,13 (1,04) | 5,25 (1,02) |
| HDL-C (mmol/L) | 1,18 (0,32) | 1,17 (0,33) | 1,16 (0,33) | 1,17 (0,30) | 1,23 (0,34) | 1,22 (0,36) |
| LDL-C (mmol/L) | 3,61 (0,52) | 3,41 (0,90) | 3,23 (0,80) | 3,38 (0,74) | 3,03 (0,81) | 3,21 (0,69) |
| Glucose (mmol/L) | 5,89 (1,70) | 5,70 (1,19) | 5,51 (1,01) | 5,46 (0,93) | 5,35 (0,69) | 5,50 (0,68) |
| HbA1c (mmol/mol) | 0,41 (0,10) | 0,41 (0,11) | 0,39 (0,08) | 0,37 (0,05) | 0,38 (0,06) | 0,37 (0,08) |
| HOMA-IR | 3,63 (2,33) | 3,06 (1,96) | 4,14 (11,12) | 3,13 (4,26) | 2,47 (1,81) | 2,67 (2,15) |

Cells contain Mean (SD)

BMI: Body Max Index; SBP: Systolic Blood Pressure; DBP: Diastolic Blood Pressure; GPT: Glutamic-Pyruvic Transaminase; GGT: gamma-glutamyl transferase; GOT: Glutamic-Oxalacetic Transaminase; TGL: Triglycerides; Chol-T: Total Cholesterol; HDL-C: High-Density Lipoprotein; LDL-C: Low-Density Lipoprotein; HbA1c: Glycated Hemoglobin; HOMA-IR: Homeostatic Model Assessment for Insulin Resistance.

**Appendix SA. Diet composition and Adherence Form.**

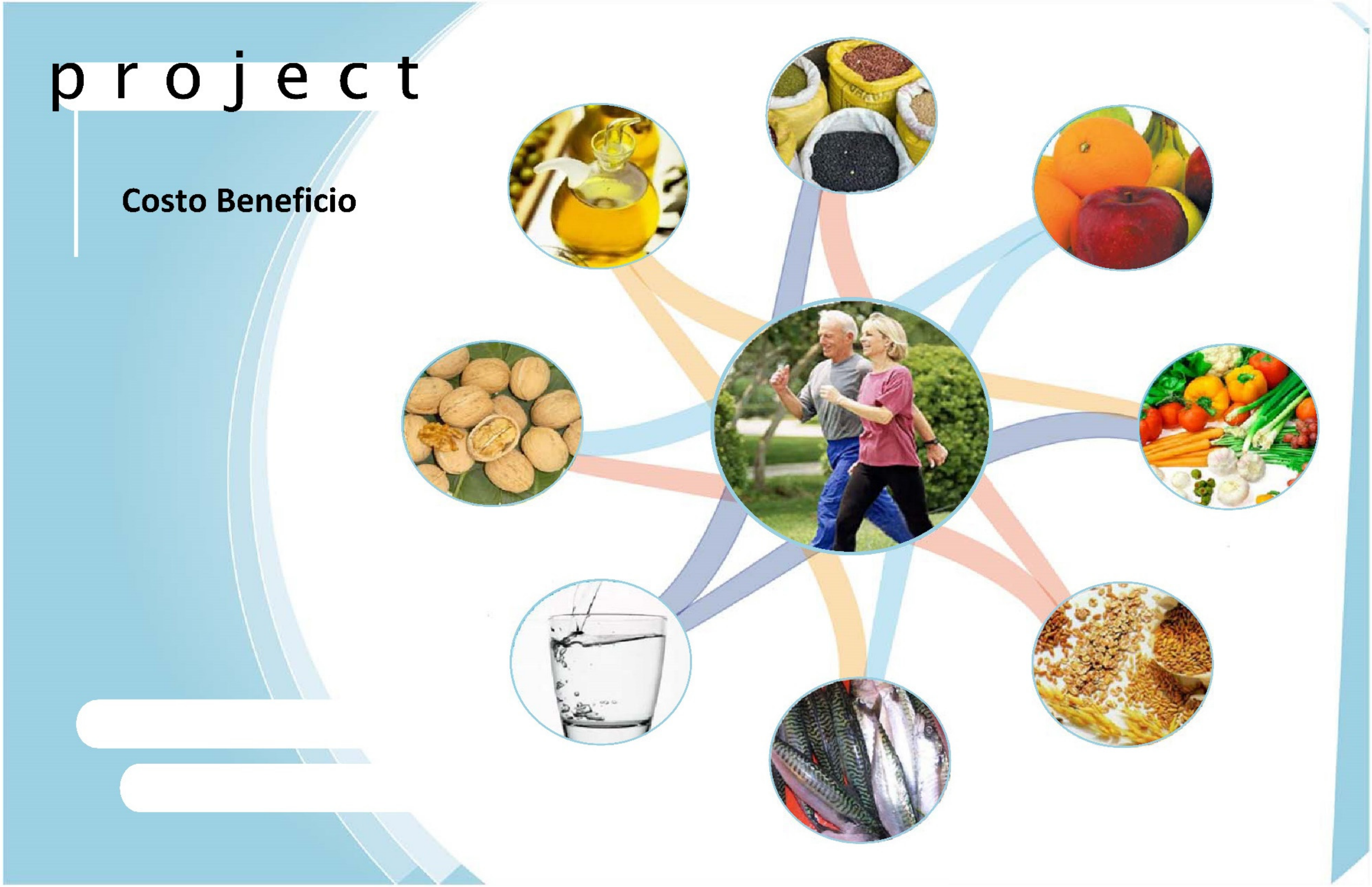


*Eat healthy*

*Adopt a correct lifestyle*

# Introduction

Your health is in your hands; you build it each day when you go to the supermarket and choose one food rather than another one, when you cook a lunch or a dinner. In these moments you do dietary choices which influence your health. A wrong diet can determine the appearance of risk factors for cardiovascular diseases and cancer. One of the steps of these diseases is the Non-alcoholic Fatty Liver Disease (NAFLD) (fat liver).

The Laboratory of Epidemiology and Biostatistics of the IRCCS Saverio de Bellis from Castellana Grotte (Bari) has started up the research project "Analisi Costo-Beneficio di un interveto basato sulla Dieta Mediterranea e l'attività fisica in pazienti diabetic e steatosici" to evaluate the efficacy of the diet and physical activity.

So, we invite you to participate in this project which will help us to understand the efficacy of the diet. Dietary advice described in the following pages are those of Low Glycemic Index Mediterranean Diet and we ask you to follow them. Furthermore, we ask you to record your daily diet in this dietary record as long as the duration of the study. This will permit us to measure adherence level to the diet we gave you.

# WATCH THE SEMAPHORE!

## CHECK YOUR DIETARY CHOICES

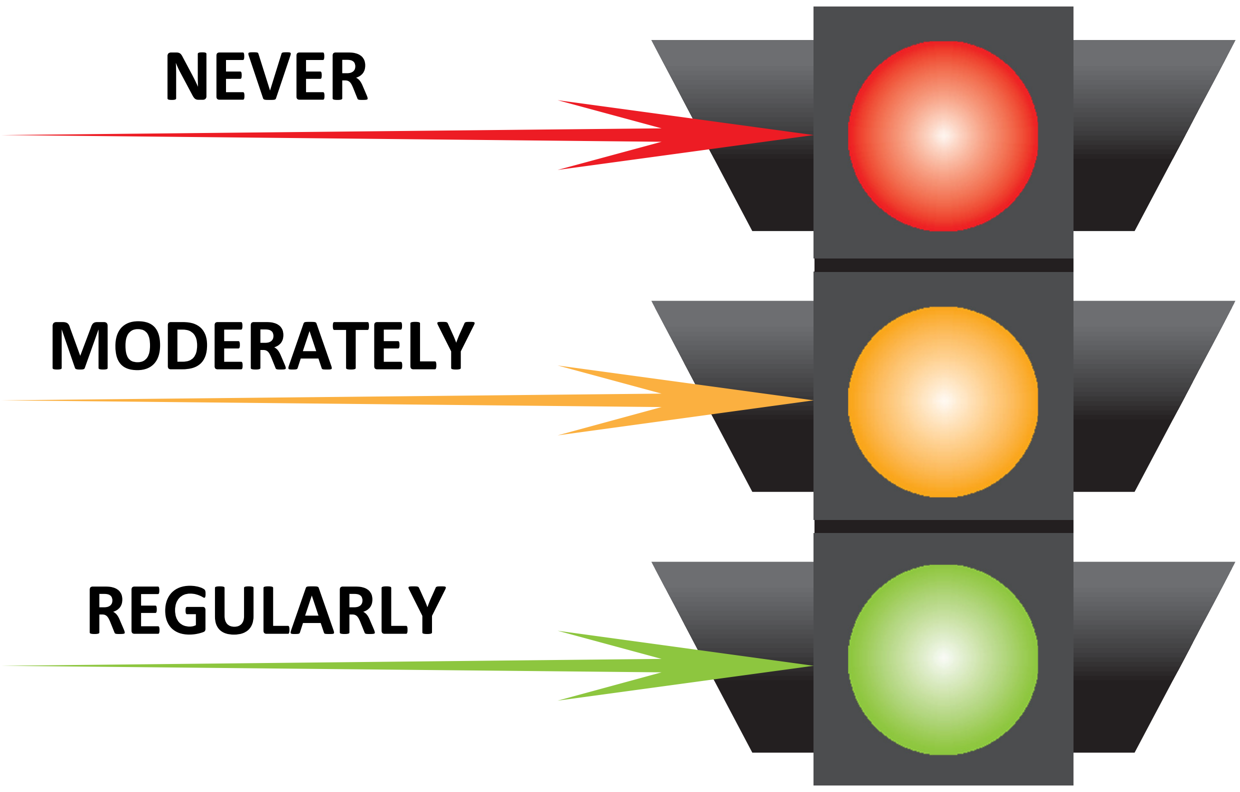


Foods choice and the frequency of their consumption is fundamental to set a correct dietary programm, which day after day should be followed by everyone to keep a satisfying state of health. To direct foods choice towards those which best represent the **LOW GLYCAEMIC INDEX**

**MEDITERRANEAN DIET**, we marked with **GREEN** colour the foods to consume regularly, with **YELLOW** those to consume moderately and with **RED** those to avoid completely.

## REGULARLY

1- Raw vegetables (lettuce, tomatoes, cucumbers, celery, carrots, radishes, etc.)

2- Steamed or boiled vegetables (beets, turnips, chicory, cauliflower, broccoli, etc.)

45- Dried pulses without something else (lentils, chickpeas, beans, broad beans, soybeans)

46- Own fresh vegetables without something else (peas, beans, green beans)

12- Whole wheat pasta with pulses

13- Whole wheat pasta with vegetables

18- Brown rice with pulses

19- Brown rice with vegetables

42- Fish (anchovies, sardines, mackerel, etc.)

40- Molluscs and crustaceans

43- Cod, swordfish, fresh tuna

66- Bass bream salmon

44- Canned tuna

28- Extra virgin olive oil (raw)

5- Unsweetened fresh fruit (apples, pears, oranges, grapefruit, kiwi, peaches, etc.)

8- Unsweetened nuts (walnuts, almonds)

65- Coffee (without sugar or with artificial sweetener)

## MODERATELY

| MODERATELY | max |
|---|---|
| 30- Milk and yogurt | 1 time/daily |
| 56- Whole wheat biscuits | 1 time/daily (3 biscuits) |
| 31- Cheese (parmigiano, cheese, etc.) | 2 times/weekly |
| 32- Dairy products (cheese, smoked cheese, cottage cheese) | 2 times/weekly |
| 33- Eggs | 2 time/weekly (1 egg) |
| 34- White meat (chicken, turkey, rabbit) | 2 times/weekly |
| 25- Potatoes (boiled only) | 1 time/weekly |
| 22- Whole wheat bread | 2 times/daily (a slice) |
| 15- Whole wheat pasta with simple sauce | 2 times/weekly (max 80 gr) |
| 4- Sugary fruit (bananas, persimmons, grapes) | 1 time/daily (max 1 piece) |
| 62- Dense pure honey | 1 time/daily (a teaspoon) |
| 49-wine | 2 times/daily (a glass |

## NEVER

11- Not whole wheat pasta
17- Not whole wheat rice 21-
White bread

53- Crackers, pretzels, bread sticks, bread and breakfast cereals

47- Pizza

35- Red meat (beef, veal, pork, horse, etc.)
36- Canned meat
37- Stew
38- Sausages (raw or cooked ham, dried beef, bacon, salami, mortadella, etc.) 41-
Farmed fish
26- French fries or baked
67- Fry

29- Butter, margarine, mayonnaise, cream 9-
Fruit in syrup and candied fruit

7- Sweet dried fruit (dried figs, dried dates, prunes, raisins)

63- Sugar

60- Candies and chocolates

61- Jams

57- Cakes, sweets, pastries

58- Croissants, pastries and snacks

54- Dry biscuits, toasts

55- Biscuits, shortbread

59- Ice-cream and water ice

51- Alcoholic beverages and spirits (brandy, grappa, liqueurs, whiskey)

50- Carbonated/Non carbonated soft drinks (orange juice, coca-cola, fruit juice, etc.)

# Your Dietary Record

## Instructions

- It's fundamental to complete the Dietary Record each day immediately after the meal or as soon as possible.
- The Dietry Record is composed of a series of pages, one for each week. In each weekly page, days from Monday to Sunday are divided in breakfast, lunch, dinner and snack.
- Record all the foods you eat and what you drink during the day (Sunday and holydays included).
- Registration consists in taking note, in correspondence to the meal, the identification number of the food/drink or of the food group consumed belonging to the list we enclosed. If the food you ate isn't included in the list, just write its name in the place reserved to the corresponding meal.

## Example:

Here below it is shown an hypothetical day of Mr Rossi's Dietary Record.

After each meal Mr Rossi registered what he ate and drank, so he wrote in the box of the specific meal the correponding number of the food he consumed (by checking the foods list).

At breakfast he drank a cup of milk and coffee without sugar and he ate an apple.

| | BREAKFAST | Snack | LUNCH | Snack | DINNER | Snack | PHYSICAL ACTIVITY |
|---|---|---|---|---|---|---|---|
| MONDAY | 30+65<br>5 | | | | | | ☐ None<br>☐ Walk<br>☐ Housework<br>☐ Training<br>☐ Other (specify what) |

At lunch he had a plate of lentils (without pasta) seasoned with raw extra-virgin olive oil, a cod, a plate of raw unseasoned vegetables and he drank two glasses of wine and a glass of natural water.

| | BREAKFAST | Snack | LUNCH | Snack | DINNER | Snack | PHYSICAL ACTIVITY |
|---|---|---|---|---|---|---|---|
| MONDAY | | | 45 + 28<br>43, 1<br>49 + 49<br>52 | | | | ☐ None<br>☐ Walk<br>☐ Housework<br>☐ Training<br>☐ Other (specify what)<br>__________ |

In the afternoon he had an apple and drank a coffee without sugar.

| | BREAKFAST | Snack | LUNCH | Snack | DINNER | Snack | PHYSICAL ACTIVITY |
|---|---|---|---|---|---|---|---|
| MONDAY | | | | 5<br>65 | | | ☐ None<br>☐ Walk<br>☐ Housework<br>☐ Training<br>☐ Other (specify what)<br>__________ |

At dinner he had a plate of minestrone soup with pasta and seasoned with raw extra-virgin olive oil, a slice of whole bread, a pear and drank two glasses of natural water.

| | BREAKFAST | Snack | LUNCH | Snack | DINNER | Snack | PHYSICAL ACTIVITY |
|---|---|---|---|---|---|---|---|
| MONDAY | 30 + 65<br>5 | | 45 + 28<br>43, 1<br>49 + 49<br>52 | 5<br>65 | Minestrone soup<br>+ 28<br>22<br>52 + 52 | | ☐ None<br>☒ Walk<br>☐ Housework<br>☐ Training<br>☐ Other (specify what)<br>__________ |

In the last column, referred to the physical activity carried out during the day, he put a cross in correspondence to the entry "walk".

SURNAME ______________________ LAST NAME ______________________ CODE ______________

MONTH ______________________ WEEK from ______________________ to ______________

| | BREAKFAST | Snack | LUNCH | Snack | DINNER | Snack | PHYSICAL ACTIVITY |
|---|---|---|---|---|---|---|---|
| Monday | | | | | | | ☐ None<br>☐ Walk<br>☐ Housework<br>☐ Training<br>☐ Other (specify what) ______ |
| Tuesday | | | | | | | ☐ None<br>☐ Walk<br>☐ Housework<br>☐ Training<br>☐ Other (specify what) ______ |
| Wednesday | | | | | | | ☐ None<br>☐ Walk<br>☐ Housework<br>☐ Training<br>☐ Other (specify what) ______ |
| Thursday | | | | | | | ☐ None<br>☐ Walk<br>☐ Housework<br>☐ Training<br>☐ Other (specify what) ______ |
| Friday | | | | | | | ☐ None<br>☐ Walk<br>☐ Housework<br>☐ Training<br>☐ Other (specify what) ______ |
| Saturday | | | | | | | ☐ None<br>☐ Walk<br>☐ Housework<br>☐ Training<br>☐ Other (specify what) ______ |

| | | | | | | | |
|---|---|---|---|---|---|---|---|
| Sunday | | | | | | | ☐ None<br>☐ Walk<br>☐ Housework<br>☐ Training<br>☐ Other (specify what) |
